# Counter-intuitive penetration of droplets into hydrophobic gaps in theory and experiment

**DOI:** 10.1038/s41598-023-43138-2

**Published:** 2023-10-02

**Authors:** Daniel Hagg, Alexander Eifert, Aaron Dörr, Francisco Bodziony, Holger Marschall

**Affiliations:** 1 Robert Bosch GmbH, Corporate Research, 71272 Renningen, Germany; 2 Mathematical Modeling and Analysis, Technical University of Darmstadt, 64287 Darmstadt, Germany

**Keywords:** Mechanical engineering, Fluid dynamics

## Abstract

Droplets that spontaneously penetrate a gap between two hydrophobic surfaces without any external stimulus seems counterintuitive. However, in this work we show that it can be energetically favorable for a droplet to penetrate a gap formed by two hydrophobic or in some cases even superhydrophobic surfaces. For this purpose, we derived an analytical equation to calculate the change in Helmholtz free energy of a droplet penetrating a hydrophobic gap. The derived equation solely depends on the gap width, the droplet volume and the contact angle on the gap walls, and predicts whether a droplet penetrates a hydrophobic gap or not. Additionally, numerical simulations were conducted to provide insights into the gradual change in Helmholtz free energy during the process of penetration and to validate the analytical approach. A series of experiments with a hydrophobic gap having an advancing contact angle of $115^\circ$, a droplet volume of about 10 $\mu$L and different gap widths confirmed the theoretical predictions. Limits and possible deviations between the analytical solution, the simulation and the experiments are presented and discussed.

## Introduction

In the past, penetration of water into cylindrical capillaries and microfluidic channels has already been intensively studied and showed that hydrophobization of surfaces inside a capillary hinders water penetration. Considering the example of a cylindrical capillary placed at the surface of a water basin, a contact angle of more than $90^\circ$ is sufficient to prevent spontaneous penetration. Based on this experience with cylindrical capillaries, one could conclude that hydrophobic gaps generally prevent the penetration of water droplets. However, we will show that this is not the case.

In 1988, Marmur already showed that spontaneous penetration of water even into hydrophobic cylindrical capillaries is possible for small droplets^1^. He developed an analytical approximation for the kinetics of droplets penetrating capillaries and showed that spontaneous penetration is possible for contact angles above $90^\circ$. His approach is based on evaluating the Laplace pressure inside the droplet, that ultimately pushes the droplet into the hydrophobic capillary. By this approach, he predicted complete penetration of droplets for contact angles up to about $114^\circ$. Following that, several other researchers investigated the penetration of droplets in cylindrical capillaries experimentally^2–7^, using molecular dynamics simulations^8–11^, phase-field simulations^12,13^ and Volume-of-Fluid simulations^14^. The findings have further been used to measure surface tensions from liquid marbles^15,16^ and to gain insights in the process of pore penetration^17–21^. Marmur also extended his approach to radial capillaries^22^, which led to further simulative^23^ and experimental^24^ investigations.

Piroird et al. suggested and experimentally validated an analytical model for droplet extraction from capillaries based on the Laplace pressure^25^. Schebarchov & Hendy used a different approach for cylindrical capillaries by calculating the total surface energy to determine penetration and validated the model with molecular dynamic simulations^26,27^. A further similar approach was used by Bormashenko et al. to calculate the energy barrier between Cassie-Baxter and Wenzel state transition^28–30^. However, Bormashenko et al. considered droplets which are several magnitudes bigger than the roughness of the structure and the deformation of the droplet was neglected.

**Figure 1:**
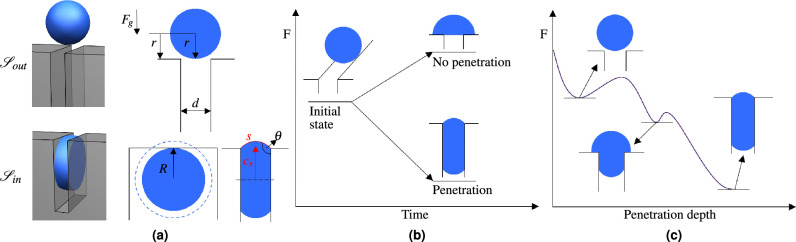
Visualization of the analytical approach and schematic trend of the Helmholtz free energy. In (**a**) the two states assumed for the analytical approach are visualized in 3D (left) and 2D (right). State ${\mathscr {S}}_{out}$ is defined as a spherical drop with radius *r*, at the entry of the gap. State ${\mathscr {S}}_{in}$ is defined as a cylinder inside the gap with a radius *R*, extended by a surrounding circular segment. The circular segment with line centroid $c_s$ and arc length *s* results from the surface contact angle $\theta$. In both states the gap width *d*, contact angle $\theta$ and droplet volume *V* are the same. In (**b**) two possible end states after a droplet impact are visualized. In case of a higher Helmholtz free energy for ${\mathscr {S}}_{in}$ compared to ${\mathscr {S}}_{out}$, no penetration is assumed and penetration otherwise. A potential schematic trend of the Helmholtz free energy is plotted in (**c**), showing an energy barrier at the beginning and a local minimum between both states. For the analytical approach the states ${\mathscr {S}}_{out}$ and ${\mathscr {S}}_{in}$ are considered.

While there has been quite some research on droplets penetrating hydrophobic cylindrical capillaries and structured surfaces, the phenomenon of penetration into a hydrophobic gap between two parallel plates as visualized in Fig. 1 (a) seems to be quite unexplored. We could only find an investigation for droplet penetration into strong hydrophilic gaps^31,32^. To close this “gap” we present here our results on droplet penetration into hydrophobic gaps between two parallel plates, using three different types of investigations:

- Analytical approach with a Helmholtz free energy
- Direct numerical phase-field simulation
- Experimental validation series

We first introduce an analytical approach, based on the Helmholtz free energy, to predict whether droplets penetrate a hydrophobic gap between two parallel plates. Using that approach we derive an equation for the change of the Helmholtz free energy depending solely on the droplet volume *V*, the contact angle $\theta$ and the gap width *d* (see Fig. 1a). In contrast to the approaches for capillary penetration referenced above, the deformations of a droplet penetrating a gap between two parallel plates are more complex and cannot be described using a two-dimensional axisymmetric plane. In order to verify our approach, the individual terms of our derived analytical equation (Eq. 4) are compared with the results of a numerical phase-field simulation. Additionally, the simulation of spontaneous droplet penetration allows an investigation and discussion of the gradual change of each free energy term in Eq. (4) during the penetration process. Further simulations show a good agreement with the analytical predictions for a droplet volume of 10 $\mu$L, contact angles of $115^\circ$, $130^\circ$ and $145^\circ$ and varying gap widths. The analytical approach is supported and validated by a series of experimental results with varying gap widths, an advancing contact angle of $115^\circ$ and a droplet volume of about 10 $\mu$L. Through our investigation with simulations and experiments we can show that the chosen approach performs very well and is rather conservative in predicting the prevention of droplet penetration into a gap. Contradicting the intuition, we find that hydrophobic gaps, and in certain conditions (contact angle, gap size and droplet volume) even superhydrophobic gaps, are not safe from droplet penetration. As hydrophobic and super-hydrophobic surfaces are often used for anti-wetting, anti-corrosion or anti-icing purposes, the penetration of water into gaps between such surfaces shall be prevented. With our approach it is possible to predict the penetration of droplets for a lot of different parameters in a short time and to adjust them in such a way that the penetration is prevented.

## Analytical approach

For our analytical approach we used the thermodynamic free energy to determine if droplet penetration is energetically favorable. With a constant volume and a constant number of particles under isothermal conditions the change in free energy is given by the Helmholtz free energy as

1 $$\begin{aligned} \text {d}F = \sum _{i}(\gamma _i \text {d} A_i) + \text {d}U_{grav} \text {.} \end{aligned}$$

For each surface *i*, the change of its free surface energy is determined by the change of its surface area multiplied with the corresponding surface energy $\gamma _i$. The change in gravitational energy $\text {d}U_{grav} = - \mathbf {F_G} \cdot \text {d}{\textbf{x}}$ is given by the gravitational force $\mathbf {F_G}$ multiplied with the change in height of the droplets center of mass $\text {d}{\textbf{x}}$.

In order to minimize the free energy, the drop will penetrate the gap as long as $\text {d}F < 0$. Owing to the complex deformations that are taking place during the penetration of the droplet, a continuous analytical description of the surfaces and the change in the Helmholtz free energy $\text {d}F$ is not possible. Therefore, in this paper we compare the energy at two separate states, namely ${\mathscr {S}}_{out}$ and ${\mathscr {S}}_{in}$, visualized in Fig. 1a. State ${\mathscr {S}}_{out}$ describes the droplet outside of the gap and is defined by a sphere with a radius *r*. Here the centre of the sphere is placed in the middle of the gap in a distance of *r* from the gap entry. ${\mathscr {S}}_{in}$ describes the droplets state inside the gap. The wetted solid area in ${\mathscr {S}}_{in}$ is assumed to be circular with radius *R*. Depending on the contact angle $\theta$, the curvature of the water-air surface is adjusted accordingly, as visualized in red in Fig. 1 (a). The length of the curved line is given by *s* and the line centroid is given by $c_s$. The droplets centre of mass is located at a distance of *R* from the gap entry. The droplet volume is denoted by *V*, the gap width by *d* and the contact angle by $\theta$.

With these two states we are able to calculate the Helmholtz free energy in the state ${\mathscr {S}}_{out}$ before it impacts the gap and in the state ${\mathscr {S}}_{in}$, where it has completely penetrated the gap. If the Helmholtz free energy is higher in the state ${\mathscr {S}}_{in}$ inside the gap than in the initial state ${\mathscr {S}}_{out}$, the penetration would lead to a rise in free energy. This rise in free energy would be thermodynamically unfavorable and, therefore, no penetration is predicted. This case is visualized in the upper part of Fig. 1 (b) labeled by “no penetration”. However, if the state ${\mathscr {S}}_{in}$ inside the gap has a lower Helmholtz free energy than in state ${\mathscr {S}}_{out}$, it is energetically favourable. In this case penetration is predicted as shown in the “penetration” case in Fig. 1 (b).

Between the initial impact and the full penetration, the droplet passes through various intermediate states. These intermediate states are not known in the current approach, which renders a continuous calculation of the Helmholtz free energy impossible. In Fig. 1c, a potential schematic trend of the Helmholtz free energy is plotted to discuss possible deviations due to the missing continuous description. In the plotted trend, the Helmholtz free energy for the state ${\mathscr {S}}_{in}$ is lower than for the state ${\mathscr {S}}_{out}$ and the comparison between the two states would predict droplet penetration. However, temporary increases in the Helmholtz free energy could prevent the droplet from penetrating the gap. Figure 1 (c) shows a possible temporary increase in the Helmholtz free energy starting right from the initial state. Such an increase could act as an energy barrier and would prevent the droplet from even starting to penetrate the gap. Nevertheless, small mechanical vibrations of the water surface or an initial droplet speed could be enough to overcome the energy barrier. Once the initial barrier is overcome, the droplet could achieve a next possible local minimum between the two assumed states. This local minimum would lead to a metastable state of partial penetration, where the droplet could cease to move. Schebarchov & Hendy has already described a local energy minimum for partial penetration of droplets into hydrophobic cylindrical capillaries^26^. Regardless of whether these local energy barriers are present or not, it is uncertain if they are relevant to real use cases due to mechanical vibrations or an initial droplet velocity.

To calculate the Helmholtz free energy in the two states ${\mathscr {S}}_{out}$ and ${\mathscr {S}}_{in}$, there are three different types of surfaces to be considered, namely the liquid-gas (LG), the solid-gas (SG) and the solid-liquid (SL) surface with the respective surface energies $\gamma _{LG}$, $\gamma _{SG}$ and $\gamma _{SL}$. The energy difference $\Delta F= F_{in} - F_{out}$ is given by integrating Eq. (1) from ${\mathscr {S}}_{out}$ to ${\mathscr {S}}_{in}$

2 $$\begin{aligned} \Delta F = \int _{A_{{LG}_{out}}}^{A_{{LG}_{in}}} \gamma _{LG} \text {d}A_{LG} + \int _{A_{{SL}_{out}}}^{A_{{SL}_{in}}}\gamma _{SL} \text {d}A_{SL} +\int _{A_{{SG}_{out}}}^{A_{{SG}_{in}}}\gamma _{SG} \text {d}A_{SG} - \int _{h_{out}}^{h_{in}} \mathbf {F_G} \cdot \text {d}{\textbf{x}} \text { .} \end{aligned}$$

With the gravitational acceleration *g*, the density difference $\Delta \rho =\rho _{water}-\rho _{air}$ and constant surface energy densities, integration results in

3 $$\begin{aligned} \Delta F = [\gamma _{LG} A_{LG} + \gamma _{SL} A_{SL} +\gamma _{SG} A_{SG} ]_{in} - [\gamma _{LG} A_{LG} +\gamma _{SG} A_{SG}+ \gamma _{SL} A_{SL} ]_{out} - \Delta \rho V g \Delta h \text {.} \end{aligned}$$

Here $\Delta h = h_{out} - h_{in}$ describes the height difference of the droplets center of mass. It is given by $\Delta h = (r+R)$ for a vertical case and by $\Delta h = 0$ for a horizontal case. Considering a constant solid surface ($A_{SG} + A_{SL} = A_S = const$) and applying the Young equation^33^ $\gamma _{LG} cos(\theta )= \gamma _{SG} - \gamma _{SL}$, Eq. (3) can be simplified to

4 $$\begin{aligned} \Delta F = \gamma _{LG} (A_{{LG}_{in}}-A_{{LG}_{out}}) - \gamma _{LG} cos(\theta ) (A_{{SL}_{in}}-A_{{SL}_{out}}) - \Delta \rho V g \Delta h \text {.} \end{aligned}$$

As described above the three input parameters, droplet volume *V*, gap width *d* and contact angle $\theta$ stay constant in both states ${\mathscr {S}}_{in}$ and ${\mathscr {S}}_{out}$. For ${\mathscr {S}}_{out}$, the radius $r=r(V)$ can be calculated from the droplet volume and, therefore, the liquid-gas and solid-liquid areas are:

5 $$\begin{aligned} A_{{LG}_{out}} = 4 \pi r^2(V) \text {,\hspace{0.4cm}}A_{SL_{out}}=0 \end{aligned}$$

All variables used here are visualized in Fig. 1a, but for the sake of brevity the full calculations are carried out in the "Methods" section. In short, the radius *R* can be calculated by setting up an equation for the volume using Guldins second rule^34^ and solving it for *R* resulting in $R=R(\theta , d, V)$. The surface $A_{{LG}_{in}}$ can be calculated with Guldins first rule using the length of the surface line *s* and its centroid $c_s$ resulting in $s=s(\theta ,d)$ and $c_s=c_s(R,\theta ,d,V)$. With these values the areas for ${\mathscr {S}}_{in}$ can be calculated as

6 $$\begin{aligned} A_{{LG}_{in}} = 2 \pi s(\theta ,d) c_s(R,\theta ,d,V) \text {,\hspace{0.4cm}} A_{{SL}_{in}} = \pi R^2(\theta , d, V) \text {.} \end{aligned}$$

For the change in the Helmholtz free energy $\Delta F$ between two states the following calculation was derived:

7 $$\begin{aligned} \begin{aligned} \Delta F =&-\gamma _{LG} \underbrace{\frac{\pi d \left( 2\theta -\pi \right) }{ cos(\left( \theta \right) )} \left( \frac{d}{\left( 2\theta -\pi \right) } +\frac{d}{2} tan\left( \theta \right) +\left( \sqrt{\left( \frac{V}{\pi d}+ \left( \frac{d}{4 cos\left( \theta \right) ^2}\right) (\left( 2\theta -\pi \right) + sin\left( 2\theta \right) ) \left( \frac{2 d cos\left( \theta \right) ^2}{3 (\left( 2\theta -\pi \right) +sin\left( 2\theta \right) )} +\frac{d}{2} tan\left( \theta \right) \right) +\left( \frac{d(\left( 2\theta -\pi \right) + sin\left( 2\theta \right) )}{8 cos\left( \theta \right) ^2}\right) ^2\right) } -\frac{d(\left( 2\theta -\pi \right) + sin\left( 2\theta \right) )}{8 cos\left( \theta \right) ^2}\right) \right) }_{A_{{LG}_{in}}} \\&- \gamma _{LG} \underbrace{\left( 4 \pi \left( \frac{3V}{4 \pi }\right) ^{\frac{2}{3}}\right) }_{A_{{LG}_{out}}} - \gamma _{LG} cos(\theta ) \underbrace{\pi \left( \sqrt{\left( \frac{V}{\pi d}+ \left( \frac{d}{4 cos\left( \theta \right) ^2}\right) (\left( 2\theta -\pi \right) + sin\left( 2\theta \right) ) \left( \frac{2 d cos\left( \theta \right) ^2}{3 (\left( 2\theta -\pi \right) +sin\left( 2\theta \right) )} +\frac{d}{2} tan\left( \theta \right) \right) +\left( \frac{d(\left( 2\theta -\pi \right) + sin\left( 2\theta \right) )}{8 cos\left( \theta \right) ^2}\right) ^2\right) } -\frac{d(\left( 2\theta -\pi \right) + sin\left( 2\theta \right) )}{8 cos\left( \theta \right) ^2}\right) ^2}_{A_{{SL}_{in}}} \\&- \Delta \rho V g \left( \underbrace{\left( \frac{3V}{4 \pi }\right) ^{\frac{1}{3}}}_r+\underbrace{\left( \sqrt{\left( \frac{V}{\pi d}+ \left( \frac{d}{4 cos\left( \theta \right) ^2}\right) (\left( 2\theta -\pi \right) + sin\left( 2\theta \right) ) \left( \frac{2 d cos\left( \theta \right) ^2}{3 (\left( 2\theta -\pi \right) +sin\left( 2\theta \right) )} +\frac{d}{2} tan\left( \theta \right) \right) +\left( \frac{d(\left( 2\theta -\pi \right) + sin\left( 2\theta \right) )}{8 cos\left( \theta \right) ^2}\right) ^2\right) } -\frac{d(\left( 2\theta -\pi \right) + sin\left( 2\theta \right) )}{8 cos\left( \theta \right) ^2}\right) }_R \right) \end{aligned} \end{aligned}$$

Although the Eq. (7) seems to be cumbersome, the computational effort is negligible, especially compared to typical CFD simulations. This allows a fast computation of $\Delta F$ for whole sets of variables. All calculations in this paper were executed with the physical parameters given in Table 1 corresponding to water and air at 20 °C.

**Table 1:** Physical parameters used for calculations.

Property		Value
Density $[\text {kg}\,\text {m}^{-3}]$	$\rho _{\text {water}}$	998
	$\rho _{\text {air}}$	1.2
Kinematic viscosity $[\text {m}^{2}\,\text {s}^{-1}]$	$\mu _{\text {water}}$	1.002e-6
	$\mu _{\text {air}}$	1.508e-5
Surface tension $[\text {N}\,\text {m}^{-1}]$	$\gamma _{LG}$	0.072
Gravitational acceleration $[\text {m}\,\text {s}^{-2}]$	*g*	9.81

In summary, the analytical approach compares the gravitational and surface energies at two distinct points visualized in Fig. 1a. If the energy difference between these two states is positive, no penetration is predicted. Therefore, the approach allows fast approximation of water penetration into the cavity. However, a dynamic behavior during the penetration cannot be described by this method.

## Analytical results

**Figure 2:**
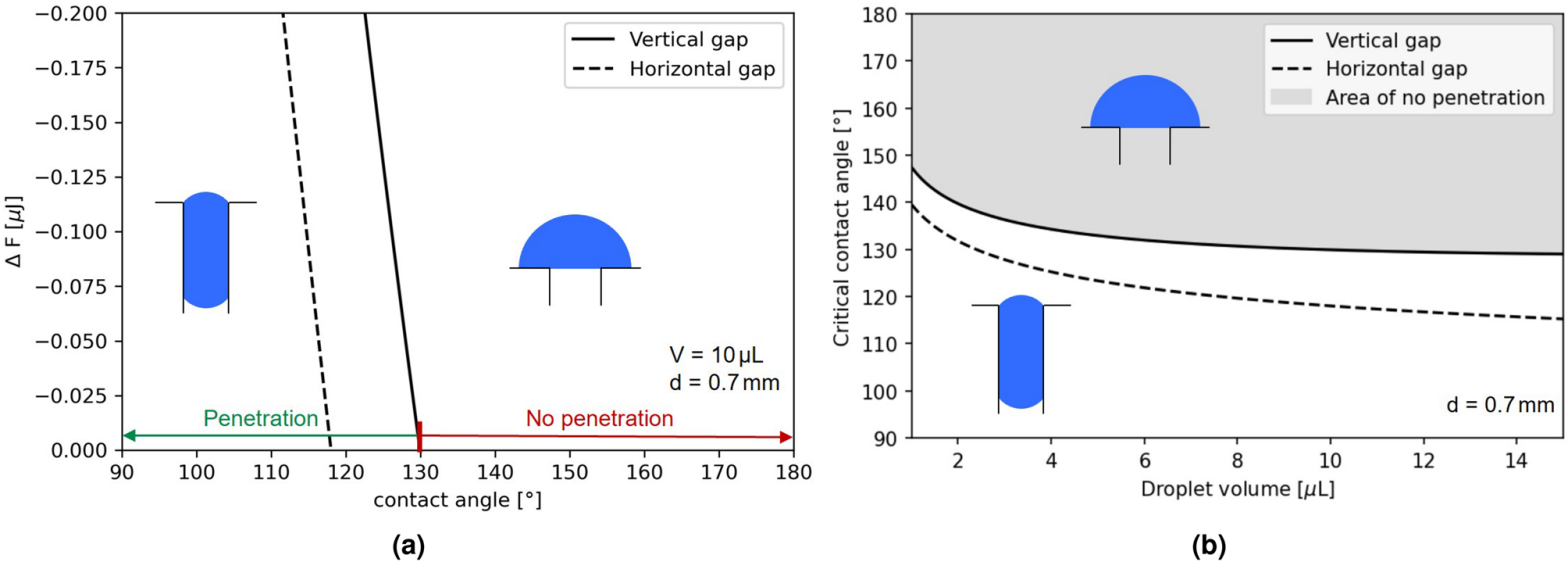
Exemplary results of an analytical calculation. In (**a**) the change of Helmholtz free energy $\Delta \text {F}$ (7) is plotted over the contact angles for a droplet volume of $V=10$ $\mu$L and a gap width of $d=0.7$ mm. The dashed line shows the results for a horizontal gap without gravitational influence, reaching $\Delta \text {F}=0$ at a critical contact angle of about $118^\circ$. The solid line shows the result for a vertical gap with gravitational influence and a critical contact angle of about $130^\circ$. For a gap width of 0.7 mm the critical contact angles with $\Delta \text {F}=0$ are determined and plotted over various droplet volumes in (**b**). Again the dashed line shows the results for a horizontal gap, whereas the solid line shows the results for a vertical gap.

In Fig. 2a, the energy difference of the Helmholtz free energy $\Delta F$ (Eq. 7) depending on the contact angle $\theta$ is plotted for a gap width of $d=0.7$ mm and a droplet volume of $V=10$ $\mu$L.

For a horizontal gap without gravitational influence, the graph crosses the x-axis at a contact angle of about $118^\circ$. This means that for any contact angle below $118^\circ$ the Helmholtz free energy is lower for state ${\mathscr {S}}_{in}$ compared to state ${\mathscr {S}}_{out}$. For contact angles higher then $118^\circ$ no penetration of the droplet is predicted. At $118^\circ$ there is a critical point with no energetic difference between ${\mathscr {S}}_{in}$ and ${\mathscr {S}}_{out}$.

For a vertical gap, where the gravity must be taken into account, penetration is predicted for even higher contact angles up to $130^\circ$ for a gap width of $d=0.7$ mm and a droplet volume of $V=10$ $\mu$L. In Fig. 2b, a critical contact angle is plotted against a droplet volume, at which the change of Helmholtz free energy is exactly zero ($\Delta F = 0$). As can be seen in Fig. 2b, with decreasing droplet volume even higher contact angles have to be achieved to prevent droplet from penetration. However, the result for this case shows that droplets can even penetrate a superhydrophobic gap, which defies the naive intuition that superhydrophobicity prevents water penetration.

## Comparison between analytical and numerical results

In the presented analytical solution, we considered only two states ${\mathscr {S}}_{in}$ and ${\mathscr {S}}_{out}$. To understand, how the Helmholtz free energy is distributed between these two states, we conducted numerical simulations using a phase-field method. For the numerical simulation the solver phaseFieldFoam was used, which is based on the phase-field method and is briefly described in the "Methods" section. In the past, the code has already been thoroughly tested for various droplet impact and wetting cases^35–39^.

For the numerical study we chose a setup with a contact angle of $\theta =115^\circ$, a droplet volume of 10 $\mu$L and a gap width of $d=0.7$ mm. In this case, analytical data and experimental data (see section "Experimental validation") suggest droplet penetration and it needs lower computational effort compared to smaller gap widths, where finer meshes are required to resolve the gap geometry. Additionally, a curvature with radius of $r=0.6$ mm measured from the experiment is implemented. This curvature in the experiment is created by the fabrication procedure and amplified by the application of an adhesive PTFE-tape over the edge. In the simulation the round edge also avoids potential numerical difficulties arising from an otherwise sharp edge. The droplet is initialized right on top of the gap as described in the analytical approach. Physical parameters used in the simulation are listed in Table 1.

**Figure 3:**
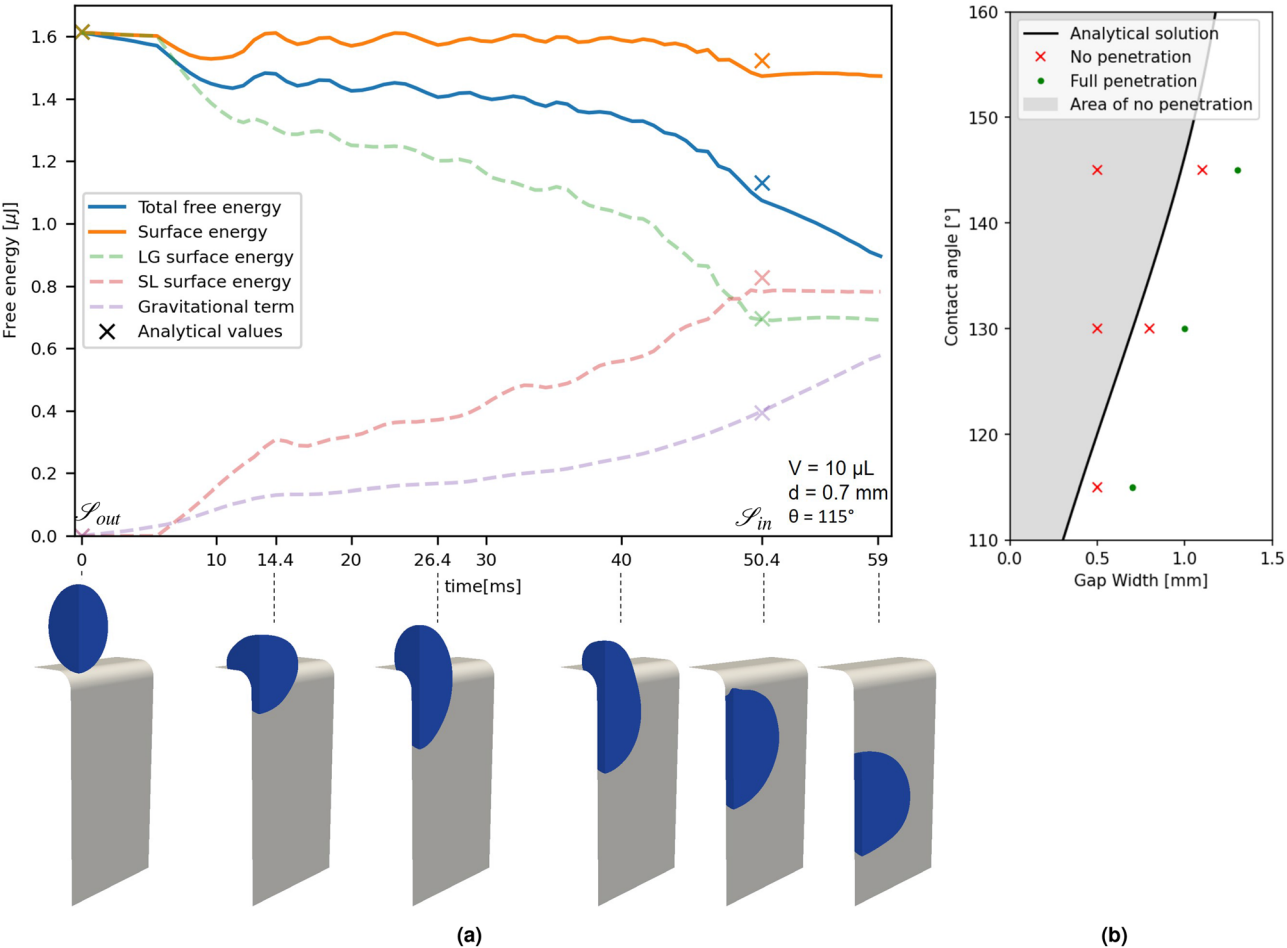
Numerical investigation of droplet penetration from phase-field simulations. In (**a**), the simulative results of a droplet with a volume of 10 $\mu$L impacting onto a gap with a width of $d=0.7$ mm and a contact angle of $\theta =115^\circ$ are shown. The droplet is initialized directly above the gap at a time of 0 ms. The edges of the gap are rounded off, using a radius of 0.6 mm to fit the experimental setup. By using two symmetry conditions, only a quarter of the simulation area is computed. In a post-process the liquid-gas surface energy, the solid-liquid surface energy and the gravitational term from Eq. (4) were calculated and plotted as dashed lines. The sum of the surface energies is plotted in orange and the total energy, including gravitational energy is plotted in blue. In addition to the continuous plots from the simulation, the analytic values for ${\mathscr {S}}_{out}$ are plotted at the initial time step $t=0$ ms. The values for ${\mathscr {S}}_{in}$ are plotted at $t=50.4$ ms, where the droplet has just completely entered the gap and no longer wets the curved corners of the gap. However, the end of the curvature in the gap is 0.6 mm lower than the upper edge of the gap, so the gravitational term in the analytical description was adjusted by the additional height of 0.6 mm. Snapshots of the simulated quarter droplet, without the air phase are shown underneath the plot to visualize the penetration process. In (**b**), the simulation results of the same setup with contact angles of $115^\circ$, $130^\circ$ and $145^\circ$ and different gap widths are marked in a graph plotting the contact angle against the gap width. Simulations where the droplets did not penetrate the gap are marked by a red “x”, whereas simulations with full droplet penetration are marked by a green dot. The bold line shows the border with no change of the Helmholtz free energy in the analytical calculation for a vertical gap.

As expected based on the analytical and experimental data, droplet penetration occurred during simulation. To investigate the progression of the energy terms in more detail, the liquid-gas area $A_{{LG}_{sim}}$, the solid-liquid area $A_{{SL}_{sim}}$ and the height of the droplets centroid $h_{sim}$ were evaluated at every time-step. The terms of the Helmholtz free energy (Eq. 4) were calculated by $F_{LG} = \gamma _{LG} A_{{LG}_{sim}}$, $F_{SL} = - \gamma _{LG} cos(\theta ) A_{{SL}_{sim}}$ and $F_g = \Delta \rho V g (h_{sim\_initial} - h_{sim})$. Additionally, the surface free energy $F_{surf} = F_{LG}+F_{SL}$ and the total Helmholtz free energy $F = F_{surf} - F_g$ were calculated and plotted in Fig. 3a.

The respective values for ${\mathscr {S}}_{out}$ and ${\mathscr {S}}_{in}$ from the analytical approach are marked with an “x”. In order to compare the surface areas of the simulation and the analytical approach, the analytical state ${\mathscr {S}}_{in}$ is marked at the time step $t=50.4$ ms, where the droplet fully penetrated the gap and does not wet rounded corners. However, the curved wall section ends 0.6 mm lower than the upper edge of the gap, so an additional height of 0.6 mm is added to calculate the gravitational term in the analytical approach. The value for ${\mathscr {S}}_{out}$ is marked at the point of initialization ($t=0$ ms).

At the initial time step, the total Helmholtz free energy is given by the liquid-gas surface energy. Since the droplet is initialized as assumed by the analytical approach, the calculated energies are the same. During the first milliseconds the droplet starts falling, resulting in an increase of the gravitational term and, therefore, a decrease of the total Helmholtz free energy (see Fig. 3a). After 6.4 ms the droplet starts wetting the wall and the solid-liquid surface energy rises. At the same time, the liquid-gas surface energy drops. Firstly, this leads to a rapid decrease of the surface energy. However, at $t \approx 9.6$ ms the surface energy reaches a minimum, because the solid-liquid energy rises faster than the liquid-gas energy decreases. The surface energy rises until $t \approx 14.4$ ms, where a maximum is observed that is nearly equal to the initial value. Furthermore, a stagnation of the gravitational term can be observed. The gravitational term is driven by the height of the droplets centroid, which nearly comes to a stop. After the first impact, the solid-liquid surface energy keeps rising, while the liquid-gas surface energy drops. This results in an oscillation of the surface energy around a more or less constant value. The gravitational term, however, starts to rise again, leading to a decrease of the total Helmholtz free energy and further penetration of the droplet. At around 40 ms the liquid-gas surface energy starts decreasing faster, leading to a decrease of the surface energy. This increases the penetration speed as can be seen from the increasing slope of the gravitational term. At $t=50.4$ ms the droplet fully penetrated the gap. Afterwards the liquid-gas and solid-liquid surface energies stay nearly constant with minor oscillations. The further decrease of the total Helmholtz free energy is attributed to the rise of the gravitational term, due to the droplet sliding down inside of the gap.

It can be seen that the simulated free energy values are in good agreement with the analytical results and the shape of the penetrated droplet fits the analytically assumed state ${\mathscr {S}}_{in}$. The strongest deviation is given for the solid-liquid surface energy which deviates just about $5.4\%$ from the analytic value.

Further simulations with contact angles of $115^\circ$, $130^\circ$ and $145^\circ$ were conducted for various gap widths. In Fig. 3b the outcomes of the respective parameter sets are marked in a graph, depending on the contact angle and the gap width. The analytical approach predicts penetration for gap widths to the right of that line and no penetration to the left of it. It can be seen that the general behavior at larger contact angles is represented correctly. However, the simulations show a slight offset and penetration is already prevented for higher gap widths, compared to the analytical solution. As shown in Fig. 3a it can be seen that at $t \approx 14.4$ ms the droplet nearly stops penetrating the gap due to a rise in surface energy based on the deformation of the droplet. For the smaller gap width of 0.5 mm and the same contact angle of $115^\circ$ a stronger deformation at the beginning of the penetration could be necessary to enter the gap, resulting in an energy barrier that prevents the penetration of the droplet (see Fig. 1c). Also viscous dissipation effects, that are only considered by the simulative approach, lead to energy losses during the deformation of the droplet and hinder the penetration process. In other words, the simulative approach also takes the dynamic behavior of the droplet penetration and therefore additional physical influences into account. While there can be several reasons for the deviation of the simulation outcomes compared to the analytical solution, we assume that they are mainly induced by an energy barrier or the viscous dissipation that are both not taken into account by the analytical approach. Numerical errors at the three-phase contact line can have a further influence and will be investigated in future work.

## Experimental validation

**Figure 4:**
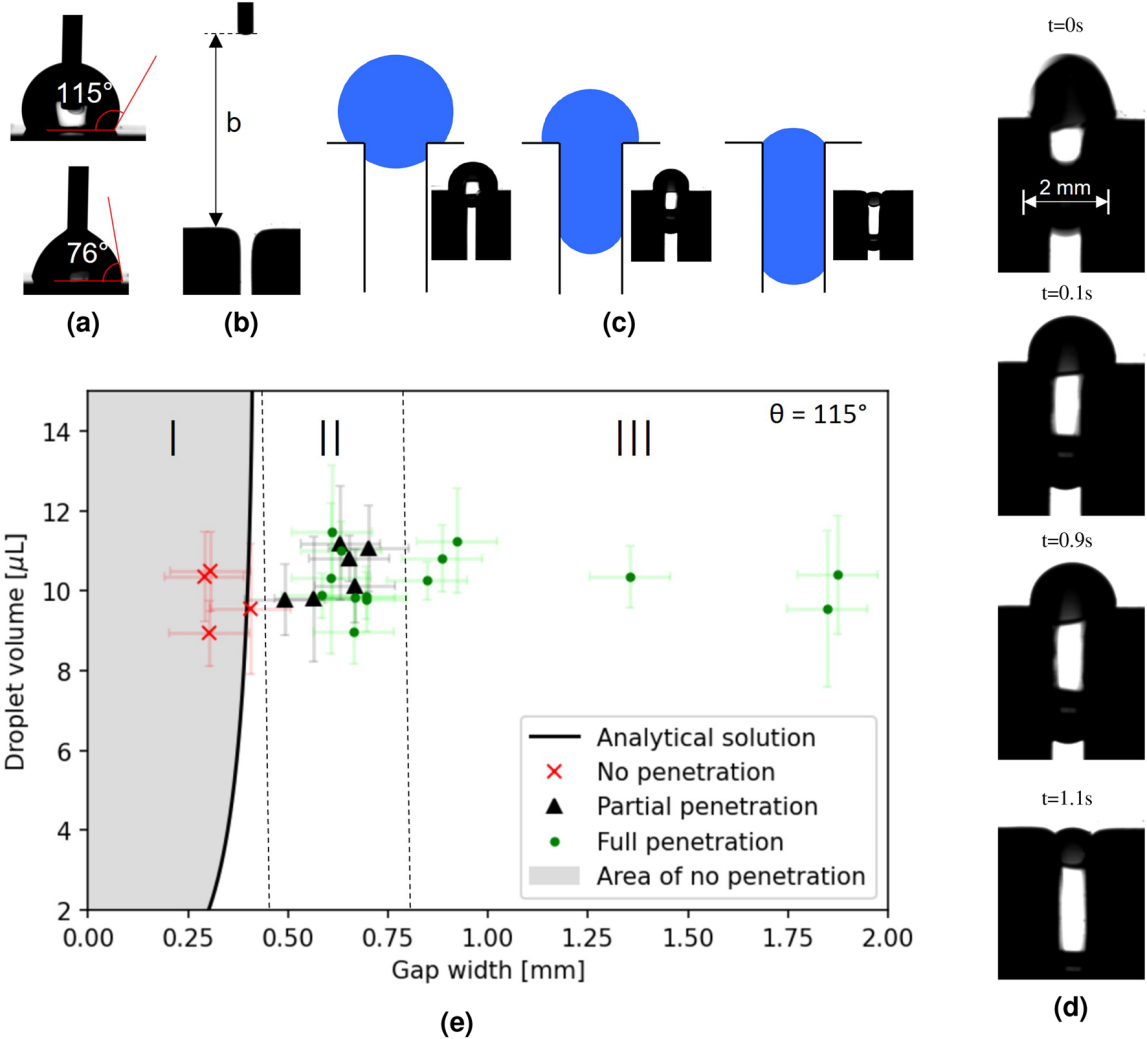
Comparison of experimental and analytical results. In (**a**) contact angle hysteresis of PTFE-tape used in experiment is shown. Original recording from Krüss DSA 100 experimental setup is depicted in (**b**), where a part of the syringe and the gap between two aluminum bodies, which were laminated with PTFE-tape, are shown. In (**c**) the three types of “no”, “partial” and “full” penetration are displayed schematically with corresponding experimental results. A special case of delayed penetration is displayed in (**d**), where a droplet of 9.8 $\mu$L impacted and firstly partially penetrated the gap. After a short amount of time ($\sim 0.8$ s), the droplet was drawn into the gap by capillary forces. In (**e**) a graph of the droplet volume against the gap width for a contact angle of $115^\circ$ is shown. The bold line in the graph represents the border where the change of the Helmholtz free energy equals zero in the analytical calculation for a vertical gap. The doted lines represent separate domains, which were observed experimentally. In domain I all experiments showed no droplet penetration. In domain II partial and full penetration were observed and in domain III all droplets fully penetrated the gap.

Experimental evaluation of droplets falling onto a hydrophobic gap were conducted to validate the analytical approach. The hydrophobic gap was shaped by two blocks of aluminium covered with an adhesive virginal Thomaplast® PTFE-tape. Multiple experiments were conducted with different distances between the two blocks. The advancing and receding contact angles of water on the PTFE surface were measured using the drop shape analyzer Krüss DSA100. The results show an advancing contact angle of $115\pm 4^\circ$ and a receding contact angle of $76\pm 4^\circ$ (see Figure 4a).

The droplet penetration experiments were also conducted on the Krüss DSA100 using the built-in syringe to create droplets of defined volume and the built-in camera to record the penetration process. During the experiments the droplets detached from the syringe at a volume of about 10 $\mu$L. The height *b* of the syringe varied between 3.8 mm and 12 mm as shown in Fig. 4b. In order to have a more accurate measurement of the droplet volume, we measured the diameters of the droplets in a post-process using images at three different time steps during the fall. In Figure 4b, a curved edge is visible at the gap entry with a measured radius of 0.6 mm. The edge curvature is induced by manufacturing of the aluminium blocks as well as by the PTFE-tape that is applied onto the edges of the blocks. Owing to the common fabrication steps in the industry the edge roundness is always present on metallic samples to some degree. The gap width was also measured by post-processing the images with a measurement error of 0.1 mm. The measurements, including the deviations due to the measurement process, are visualized in Fig. 4e.

After the droplet impact on the gap, three different droplet states were observed in the experiments. Pictures of each state, supported with schematic visualizations, are shown in Fig. 4c. The first state shows the case of “no penetration”, where the droplet shows nearly no imbibition. The second state is described as “partial penetration”, where a part of the droplet is inside the gap, but there is still a significant part of the droplet on the top. A possible reason for this metastable state was already discussed above and could be explained by a local energy minimum as visualized in Fig. 1c. However, in the experimental setup a contact angle hysteresis is clearly present. For the case of delayed penetration an analysis of the pictures showed that the dynamic receding contact angle reached contact angles down to $65^\circ$. This is not in contradiction with the measured results in the Fig. 4a, where the“static” receding contact angle was measured during a slow decrease in droplet volume. Generally, we expect different contact angles for the wetting and the dewetting processes. Dewetting a surface with a contact angle below $90^\circ$ typically consumes energy, whereas the dewetting of a surface with a hydrophobic contact angle would release energy. Therefore, the lower receding contact angle during the dewetting process on top of the gap hinders the penetration process which we indeed observed in the experiments. Consequently, the contact angle hysteresis introduces an additional energy barrier which is not considered in the numerical as well as in the analytical calculations. We believe that the contact angle hysteresis is the main reason why, compared to the simulations and analytics, there is a transition area showing partial penetration between no and full penetration (see area II in Fig. 4e). The third state is named “full penetration”, where the droplet completely enters the gap. For the full penetration a special case of “delayed penetration” was experimentally observed, shown in Fig. 4d. Here, the droplet firstly achieved metastable partial penetration stage, but after an additional 0.8 seconds the droplet was suddenly drawn into the gap completely. This was observed for a gap width of 0.7 mm and a droplet volume of 9.8 $\mu$L and is counted as full penetration. In Fig. 4e, the experimental and analytical results are plotted, depending on the gap width *d* and the droplet volume *V*. The analytical results for the critical vertical gap width are calculated for a contact angle of $115^\circ$ and plotted as a bold line. The area to the left side of the critical values, where the analytical approach predicts no penetration of the droplets, is marked in grey. The experimental results are marked by a red “x” for no penetration, a black triangle for partial penetration and a green dot for full penetration. The experimental results were divided in three domains with different observations, depending on the gap width. In domain I all droplets stayed on top of the gap after the impact, resulting in the state of no penetration. This outcome fits the analytical prediction very well. The highest gap width resulting in no penetration was observed at a value of $d=0.4$ mm. In this domain, the additional height *b* of the syringe did not have an observable impact. In domain II, covering experiments with bigger gap widths, two separate outcomes were observed. One part of the experiments showed a state of partial penetration after the impact, and the other part showed full penetration of the gap. In this domain a dependency between the syringe height and the outcome was observed. Droplets detaching from a higher syringe were more likely to completely penetrate the gap. In this case, the additional height seems to provide the necessary gravitational and, therefore, kinetic energy to overcome the metastable state of partial penetration. Another observed influence on the successful penetration in domain II was an exactly centered impact of the droplet on the middle of the gap. In domain III with gap widths larger than 0.8 mm all experiments resulted in droplets that fully penetrated the gap.

## Conclusions

In this work we showed that droplets penetrate hydrophobic and, in some cases, even superhydrophobic gaps. We derived an analytical equation to predict droplet penetration into a hydrophobic gap, depending on the droplet volume *V*, the contact angle $\theta$ and the gap width *d* (Eq. 7). Once this equation is implemented, it is easy to calculate and predict whether a droplet will penetrate a hydrophobic gap or not. The low computational effort allows predictions for large amounts of parameter variations. In the analytical equation, the change in Helmholtz free energy is calculated by assuming the two droplet states ${\mathscr {S}}_{out}$ and ${\mathscr {S}}_{in}$ (see Fig. 1a). However, the determination of the gradual change in Helmholtz free energy between these two states was not possible in the analytical description. To overcome this limitation, we conducted a numerical investigation using a phase-field method for the case of $V=10$ $\mu$L, $d=0.7$ mm and $\theta =115^\circ$. As expected, we have found that the Helmholtz free energy decrease is not evenly distributed throughout the penetration process. Slight oscillations in surface energies can be seen during the droplet penetration (see Fig. 3a). Additional simulations were carried out to validate the analytical predictions for a range of contact angles. The results were in good agreement with the analytical approach, with a slight offset due to an energy barrier during the first phase of penetration, caused by geometrical deformation of the droplet at the beginning of the penetration and viscous dissipation effects. We plan to investigate this behavior in more detail in our future work, especially for smaller gap widths, where numerical solutions are computationally expensive due to higher mesh resolutions. In addition to the numerical and analytical investigations, experiments of droplets impacting hydrophobic gaps were conducted to validate the theoretical results. These experiments confirmed that droplet penetration into hydrophobic gaps also occurs in reality, defying intuition. The results showed a very good agreement with the analytical and numerical predictions. However, in the experiments we observed not only full and no penetration, but also partial penetration of the droplets (see Fig. 4e). The main reason for this is probably the contact angle hysteresis, that leads to an energy loss during the penetration process and is currently not considered in the numerical and analytical approach. This additonal energy can also explain the height dependency of the experimental results for droplets to fully penetrate the gap in area II (see area II in Fig. 4e).

We can state that both the simulative and the experimental results show a good agreement with our analytical approach. Compared to the analytical approach, the numerical investigation takes the dynamic behavior and the geometrical deformation of the droplet into account, that showed to slightly hinder the penetration. On top of that, the experiments show an influence of the contact angle hysteresis, that also hinders penetration and induces the additional outcome of partial penetration. Conclusively, our analytical approach is in good agreement with the investigations, but rather conservative in predicting no penetration and, subsequently, of greater interest for applications in microfluidics.

## Methods

### Analytical calculations

**Figure 5:**
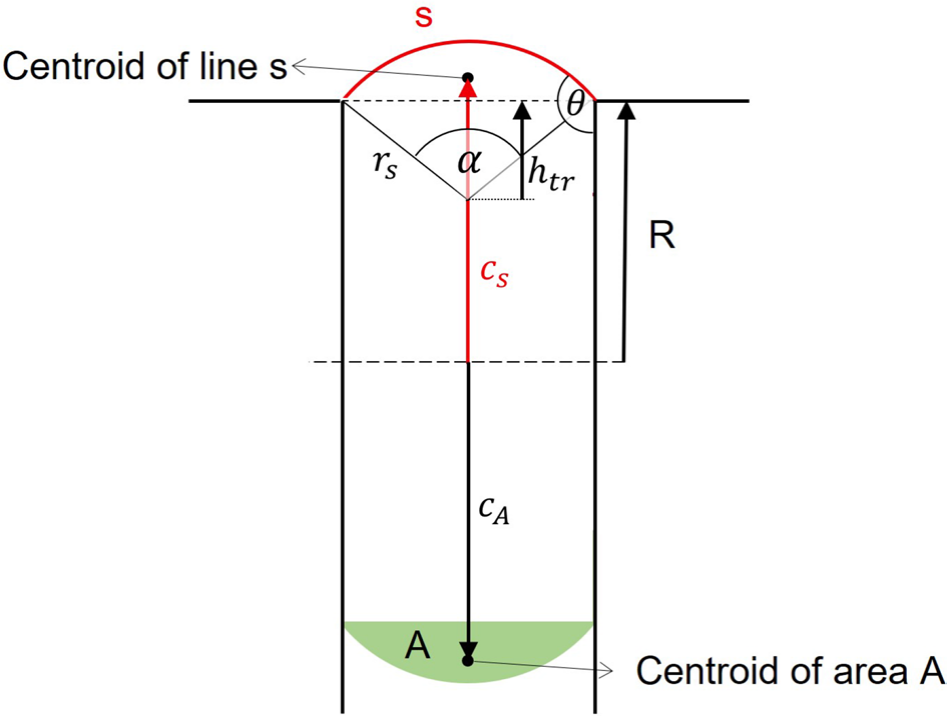
Detailed figure with all variables used in the following derivation.

In Eqs. (5, 6), the analytical approach is described, depending on the basic variables *V*, *d*, $\theta$ and the interim variables *r*, *s*, $c_s$ and *R*. In the following paragraph the equations are derived in more detail for easier comprehension. In Fig. 5 all variables, necessary to understand the derivations, are plotted.

In Eq. (5) the radius *r* of the sphere can be calculated by

8 $$\begin{aligned} r=\left( \frac{3V}{4 \pi }\right) ^{\frac{1}{3}} \text {.} \end{aligned}$$

In order to calculate the surface area $A_{{LG}_{in}}$ of the droplet inside the gap, the first Guldin rule is used^34^. Therefore the surface area can be calculated by multiplying the length of the rotationed line *s* with the path length, that the centroid $c_s$ of the line is taking during the rotation. This results in Eq. (6) ($A_{{LG}_{in}}= 2 \pi c_s \cdot s$), with the line length *s* of the surface given by

9 $$\begin{aligned} s=r_s \alpha \text { with } r_s = \frac{d}{2 sin(\frac{\alpha }{2})} \text { and } \alpha = 2\left( \theta - \frac{\pi }{2}\right) \text {.} \end{aligned}$$

The centroid of the circular arc *s* set up by the angle $\alpha$ is equal to $c_s+h_{tr}-R$ and it is given by integrating the line position over the line divided by the integral over the line

10 $$\begin{aligned} c_s+h_{tr}-R=\frac{\int _{C}x\text {d}s}{\int _{C}\text {d}s} = \frac{\int _{-\alpha /2}^{\alpha /2}r_s cos(\omega )r_s\text {d}\omega }{\int _{-\alpha /2}^{\alpha /2}r_s\text {d}\omega }=\frac{r_s sin(\alpha /2)}{\alpha /2} \text {.} \end{aligned}$$

In order to get the distance from the rotational axis up to the centroid $c_s$, the height of the drawn triangle $h_{tr}$ is subtracted and the radius R is added. This results in

11 $$\begin{aligned} c_s = \frac{r_s sin(\alpha /2)}{\alpha /2} -\underbrace{\sqrt{\left( r_s^2-\left( \frac{d}{2}\right) ^2\right) }}_{=h_{tr}}+R \text {.} \end{aligned}$$

The last sought variable is *R*, depending on $\theta$, *V* and *d*. *R* has to be calculated in such a way that the volume in ${\mathscr {S}}_{in}$ is the same as in ${\mathscr {S}}_{out}$. In order to calculate the volume in ${\mathscr {S}}_{in}$, once again Guldins second rule is used to calculate the volume resulting from the curved area *A* of the droplet. In this case the area centroid $c_A$ is needed. The centroids position $c_A$ can be calculated by integrating over the circle segment marked in green in Fig. 5, similar as for the line centroid

12 $$\begin{aligned} c_A+h_{tr}-R=\frac{\int _{F}x\text {d}A}{\int _{F}\text {d}A}= \frac{\int _{-\alpha /2}^{\alpha /2} \int _{\frac{r_s cos(\alpha /2)}{cos(\omega )}}^{r_s} r cos(\omega ) r\text {d}r\text {d}\omega }{\int _{-\alpha /2}^{\alpha /2} \int _{\frac{r_s cos(\alpha /2)}{cos(\omega )}}^{r_s}r\text {d}r \text {d}\omega } = \frac{4}{3} \frac{r_s sin^3(\alpha /2)}{(\alpha -sin(\alpha ))} \end{aligned}$$

and therefore

13 $$\begin{aligned} c_A = \frac{4}{3} \frac{r_s sin^3(\alpha /2)}{(\alpha -sin(\alpha ))} -\underbrace{\sqrt{\left( r_s^2-\left( \frac{d}{2}\right) ^2\right) }}_{=h_{tr}}+R \text {.} \end{aligned}$$

The size of the rotationed area marked in green is given by

14 $$\begin{aligned} A_s = \frac{r_s^2}{2}(\alpha - sin(\alpha )) \text {,} \end{aligned}$$

which results in a total volume of:

15 $$\begin{aligned} V = \pi R^2 d + 2\pi c_A A_s \text {.} \end{aligned}$$

The radius R can then be calculated by

16 $$\begin{aligned} R = \sqrt{\left( \frac{V}{\pi d}-\frac{2 A_s (c_A-R)}{d}+\left( \frac{A_s}{d}\right) ^2\right) }-\frac{A_s}{d} \text {.} \end{aligned}$$

### Simulation method

For the simulation the phase-field solver phaseFieldFoam was used to calculate the penetration of the water droplet. This is an in-house code developed in cooperation between the Technical University of Darmstadt and the Karlsruhe Institute of Technology based on OpenFOAM-extend^40^. The solver makes use of a Cahn-Hilliard phase-field model coupled with the Navier-Stokes equations. The simulations are set up for immiscible Newtonian fluid phases under isochoric and isothermal conditions. In the following we will give a short introduction into the phase-field method and its governing equations. For further information the reader is referred to Bodziony^39^.

The phase-field method is a diffuse interface method with a thin interfacial region in which the fluid properties vary continuously. The phases are indicated by the order parameter $C \in [-1, 1]$. The coupled Cahn–Hilliard, Navier–Stokes equations for two immiscible Newtonian fluid phases under isochoric and isothermal conditions in semi-closed formulation are given by:

17 $$\begin{aligned}{} & {} \partial _t C + \nabla \cdot (C{\textbf{u}})= - \nabla \cdot {\textbf{J}} \end{aligned}$$

18 $$\begin{aligned}{} & {} \nabla \cdot {\textbf{u}} = 0 \end{aligned}$$

19 $$\begin{aligned}{} & {} \partial _t (\rho {\textbf{u}}) + \nabla \cdot (\rho {\textbf{u}} {\textbf{u}}) = -\nabla {\tilde{p}} + \nabla \cdot \left[ \mu (\nabla {\textbf{u}} + (\nabla {\textbf{u}})^{\text {T}})\right] - \nabla \cdot (\textbf{uJ}) - \Phi \nabla C + \mathbf {f_g} \end{aligned}$$

The phase-field flux ${\textbf{J}}=-M\nabla \Phi$ is governed by the Mobility *M* and the chemical potential $\Phi$ as described by Landau and Lifshitz^41^. Following Cahn and Hilliard^42^, the chemical potential is given by the variational derivative of the total Helmholtz free energy $\Phi = \frac{\lambda }{\varepsilon ^2} \Psi '(C) - \lambda \Delta C$ with the Ginzburg-Landau potential^43^ $\Psi = \frac{1}{4}(C^2-1)^2$. The capillary width $\varepsilon$ determines the interface width in equilibrium state and is usually chosen using a Cahn number and a characteristic length scale $\varepsilon = Cn L_{char}$. The mixing energy coefficient $\lambda$ is then given by^39^ $\lambda = \frac{3}{2\sqrt{2}}\gamma \varepsilon$. The mobility is set by $M=\chi \varepsilon ^2$ with $\chi =1$ sm/kg. The modified pressure term ${\tilde{p}}$ includes parts of the well known Korteweg stress tensor term, that are accounting for interfacial capillarity. Densities and viscosities are calculated by a volumetric average to $\rho =\frac{1+C}{2}\rho _{water}- \frac{1-C}{2}\rho _{air}$ and $\mu =\frac{1+C}{2}\mu _{water}- \frac{1-C}{2}\mu _{air}$. The gravitational force is given by $\mathbf {f_g}=\rho {\textbf{g}}$ and the term $\nabla \cdot (\textbf{uJ})$ is necessary to ensure thermodynamic consistency^44^.

The phase-field boundary condition for wetted walls is given by the wetting condition

20 $$\begin{aligned} \partial _n C = \frac{cos(\theta _0)}{\sqrt{2}\varepsilon }(1-C^2) \end{aligned}$$

and a zero flux condition for the chemical potential

21 $$\begin{aligned} \partial _n \Phi = 0 \text {.} \end{aligned}$$

For pressure-velocity coupling the PIMPLE-algorithm in OpenFOAM was used with two outer correctors. For the discretization in time a second order implicit scheme was used with an adaptive time-stepping for computational efficiency. A Courant number criterion with a value of $Co = |u|_{max} \Delta t/h = 0.1$ is applied to determine the time step $\Delta t$ in combination with an upper limit for the time step size ($\Delta t_{max} = 1$ $\mu$s). In space, a second order Gauß Gamma scheme has been used.

### Supplementary Information

Supplementary Information.

## Data Availability

All data generated or analysed during this study are included in this published article and its Supplementary information files.
