# Supplementary figures and images for: Counter-intuitive penetration of droplets into hydrophobic gaps in theory and experiment
Source: Sci Rep. 2023 Oct 2;13:16518. doi: 10.1038/s41598-023-43138-2 (PMC10545836; doi:10.1038/s41598-023-43138-2)

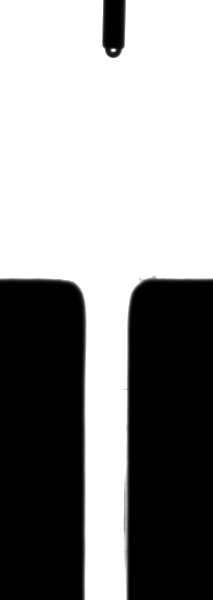

Supplement: Supplementary file 1 — Supplementary Information. [file 41598_2023_43138_MOESM1_ESM.zip › RelatedFiles_ScientificReports/Experiment/Videos/FullPenetration.gif]

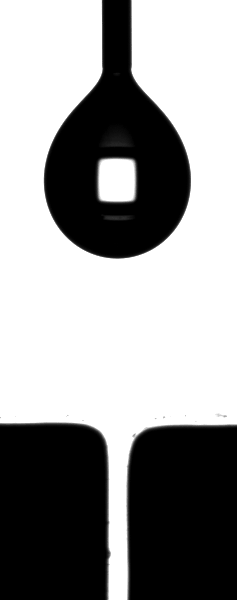

Supplement: Supplementary file 1 — Supplementary Information. [file 41598_2023_43138_MOESM1_ESM.zip › RelatedFiles_ScientificReports/Experiment/Videos/NoPenetration.gif]

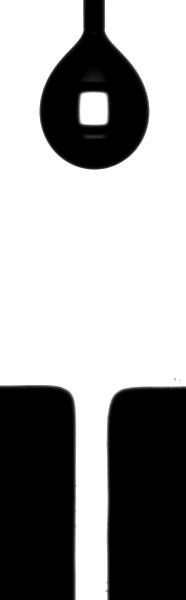

Supplement: Supplementary file 1 — Supplementary Information. [file 41598_2023_43138_MOESM1_ESM.zip › RelatedFiles_ScientificReports/Experiment/Videos/PartialPenetration.gif]

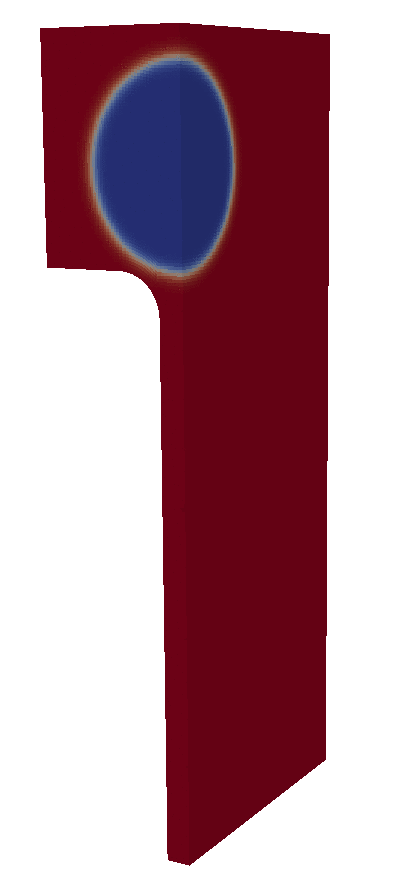

Supplement: Supplementary file 1 — Supplementary Information. [file 41598_2023_43138_MOESM1_ESM.zip › RelatedFiles_ScientificReports/Simulation/DropletSimulation.gif]
